# Magnetic particle clusters with tunable thrombus penetration for enhanced thrombolysis

**DOI:** 10.3389/fbioe.2026.1852074

**Published:** 2026-08-05

**Authors:** Yuan Lei, Zongnan Liu, Zuohong Fu, Sizhe Xiang, Wencheng Peng, Chenguo Yao, Dengfeng He, Shoulong Dong

**Affiliations:** 1 School of Electrical Engineering, Chongqing University, Chongqing, China; 2 Institute of Burn Research Southwest Hospital, Third Military Medical University (Army Medical University), Chongqing, China

**Keywords:** bioengineering, drug delivery, magnetic particle clusters, pulsed magnetic field, thrombolysis, thrombus penetration

## Abstract

Current magnetically controlled thrombolysis strategies often rely on complex micro-/nano-architectures or multimodal external field actuation, which increase technical cost and operational complexity. Developing a simple, efficient, and controllable strategy to overcome the structural barrier of thrombi therefore remains a major challenge. In this study, we propose a thrombolytic strategy based on pulsed magnetic field (PMF)-regulated magnetic particle clusters (MPCs). Using an *in vitro* thrombolysis platform, we systematically investigated the relationships between MPC-mediated penetration efficiency and key parameters, including PMF intensity and particle mass. We further characterized the time-dependent penetration behavior of MPCs in thrombi, optimized the pulse waveform, and evaluated the effect of penetration cycles on thrombolytic performance. The results show that PMF-driven MPCs can rapidly penetrate thrombi and form internal drug diffusion channels within 10 min. Compared with tPA treatment alone, thrombolysis was significantly enhanced after MPC-mediated penetration, and the enhancement increased with penetration cycle number. For 0.2 mg MPCs, the thrombolytic rate reached saturation after 15 penetration cycles and was more than twice that achieved with tPA treatment alone. Notably, under continuous perfusion at 10 mL/hr, MPCs maintained their penetration capability, and thrombolysis continued after magnetic retrieval of MPs. Collectively, these findings demonstrate that PMF-driven MPCs provide a simple, controllable strategy for enhancing intrathrombus drug transport and thrombolytic efficacy.

## 1 Introduction

Thrombotic diseases are prevalent in a number of key clinical settings, including but not limited to ischemic stroke, acute myocardial infarction, and venous thromboembolism. These diseases represent a significant pathological factor in terms of mortality and long-term dysfunction (Lutsey and Zakai, 2023; Liu et al., 2024; Wang Z. et al., 2022). Anticoagulation, pharmacologic thrombolysis, and interventional thrombectomy techniques are widely used in clinical practice. However, the achievement of rapid and stable blood flow recanalization remains a significant challenge in diverse clinical settings (Fan et al., 2022; Liu et al., 2023). This issue is closely associated with the internal thrombus structure (Zhang et al., 2022; 2023; Lin et al., 2024). Thrombus is a dense composite system dominated by fibrin networks with highly embedded platelets and red blood cells. This structure confers upon the thrombus a substantial “barrier effect”, both mechanically and in mass transfer (Tian et al., 2021). On the one hand, this structure severely limits the diffusion of thrombolytic drugs into the deeper regions of the thrombus, confining the action of the drugs to areas closer to the surface (Raynaud et al., 2021). On the other hand, it has been demonstrated that this significantly weakens the ability of mechanical perturbations to disrupt the overall thrombus structure (Wang et al., 2024). To improve local drug concentration and intrathrombus penetration, catheter-directed thrombolysis has been introduced as a representative strategy for site-specific drug delivery. However, its application is often restricted in distal, deep, or small-caliber vessels (Su et al., 2021). As a result, thrombolytic strategies that depend on diffusion or slow permeation still frequently fail to reach the thrombus core within a limited timeframe, leading to unstable or unsuccessful thrombolysis.

Recently, numerous studies have focused on developing micro/nano systems with targeted maneuverability to achieve more effective local structural perturbation and functional synergy (Chen et al., 2023; Cheng et al., 2023; Fan et al., 2025). Among these, magnetic micro/nanorobots offer unique advantages in inaccessible or space-constrained vascular environments owing to remote, non-contact actuation and precise spatial manipulation under external magnetic fields (Liu et al., 2026). In 2017, Khalil et al. proposed a mechanical friction thrombolysis method that utilizes spiral robots (Khalil et al., 2017). Spiral robots, driven by a dual rotating dipole magnetic field, have been shown to achieve directional thrombus friction and penetration in catheters, with a thrombolysis rate that is threefold that of drugs at 20–45 Hz. In 2021, Wang et al. reported the development of a spiral magnetic microcarrier that exhibits bacterial flagellum-mimetic propulsion under a rotating magnetic field (Wang Q. et al., 2022). In a rabbit carotid artery thrombus model, the penetration of the thrombus surface and formation of internal channels has been observed. Thus, it significantly enhances the diffusion efficiency of thrombolytic drugs and reduces vascular recanalization time to within 30 min. In addition to spiral propulsion systems, bioinspired magnetic microrobots (BMMs) inspired by magnetotactic bacteria have also been utilised in thrombolysis research (Xie et al., 2020). In 2020, Xie et al. reported on a bioinspired soft magnetic microrobot with rapid motion response. It achieves a maximum movement speed of 161.7 μm/s and improves targeted drug penetration under rotating magnetic field. In conclusion, magnetic micro/nanorobots have demonstrated the potential for active thrombus penetration at the functional level. However, individual magnetic micro/nano structures generally face issues such as limited driving force, complex fabrication processes, insufficient loading capacity, and inadequate imaging capabilities in practical applications. To a certain extent, these issues restrict the promotion and application of the subject in complex vascular scenarios (Sitti, 2018; Wang Q. et al., 2022; Fan et al., 2025).

In considering the inherent limitations of individual systems, the concept of magnetic particle clusters (MPCs) has gradually gained attention (Bithi et al., 2020; Haq et al., 2025; Zhou et al., 2026). In the presence of an external magnetic field, the formation of MPCs occurs through the dynamic assembly of numerous magnetic particles (MPs) via dipole–dipole interactions. From the physical perspective, such clustered configurations offer substantially higher loading capacity than individual particles, while simultaneously improving magnetic imaging contrast under actuation. Moreover, the reconfigurable morphology of MPCs facilitates effective adaptation to tortuous and narrow vascular environments (Xu et al., 2023; Wang et al., 2024; Yang et al., 2025).

The external magnetic field configurations that are applied determine the distinct behaviors and functional effects of MPCs. In early studies, the use of static magnetic fields was predominant in achieving targeted accumulation and penetration of thrombolytic drug-loaded magnetic particles at the site of thrombus (Torno et al., 2008; Anastasova et al., 2018; Chiu et al., 2019). Building on this, rotating magnetic fields were introduced to endow MPCs with new dynamic modes. Numerous studies have demonstrated that under the influence of a rotating magnetic field, MPCs have the capacity to form micro-wheels, nanomotors, or chain-like structures (Yang et al., 2023; Bao et al., 2025; Hou et al., 2025). These structures promote thrombus structural disruption by enhancing local shear stress and mechanical contact effects. For instance, the magnetic colloidal micro-wheel system developed by Tasci et al. achieved efficient self-assembly and locomotion under a 100 Hz, 9 mT rotating magnetic field. The spiral motion inhibited particle agglomeration and enabled penetration of approximately 100 μm platelet-rich clots within 5 min (Tasci et al., 2017). However, due to low magnetic field strength and weak mechanical perturbation, current approaches that rely solely on magnetically driven MPCs are insufficient for achieving effective penetration and disruption of deep thrombus layers, thereby constraining rapid recanalization (Tang et al., 2022; Li et al., 2023). Therefore, magnetic drive systems are also synergistically applied with multi-modal external excitation such as photothermal, magnetothermal, and ultrasound. Through local thermal effects, cavitation effects, or phase transition kinetics, they significantly enhance convection and micro-destruction inside the clot. This facilitates deeper penetration and thrombolysis (Zhang et al., 2021; Choi et al., 2022; Zhu et al., 2024). For example, Yu Cheng et al. adopted a dual-frequency magnetic field cooperative regulation strategy. This assembly generates mechanical forces in combination with magnetothermal effects, with the objective of penetrating thrombi (Gao et al., 2024). In 2020, Yu Gao et al. developed a magnetic microbubble system combined with ultrasound, which achieved a thrombus penetration depth of several hundred micrometers (Wang et al., 2020). Similarly, in 2023, Ruan et al. designed a composite nanomotor that, after magnetic field guidance, achieved deep thrombus penetration via near-infrared and ultrasound-triggered phase transition (Ruan et al., 2024). In 2024, Wu et al. synergistically regulated magnetic microclusters using low-intensity ultrasound and a torque magnetic field to mimic ciliary rhythmic beating, leveraging fluid disturbance and mechanical action to disrupt the thrombus fibrin network (Wu et al., 2024). Multi-modal cooperative strategies have demonstrated potential in enhancing thrombolysis efficacy. However, further research is required on complex equipment integration, technical optimization of multi-parameter coupled regulation, and compatibility issues in clinical translation.

**FIGURE 1:**
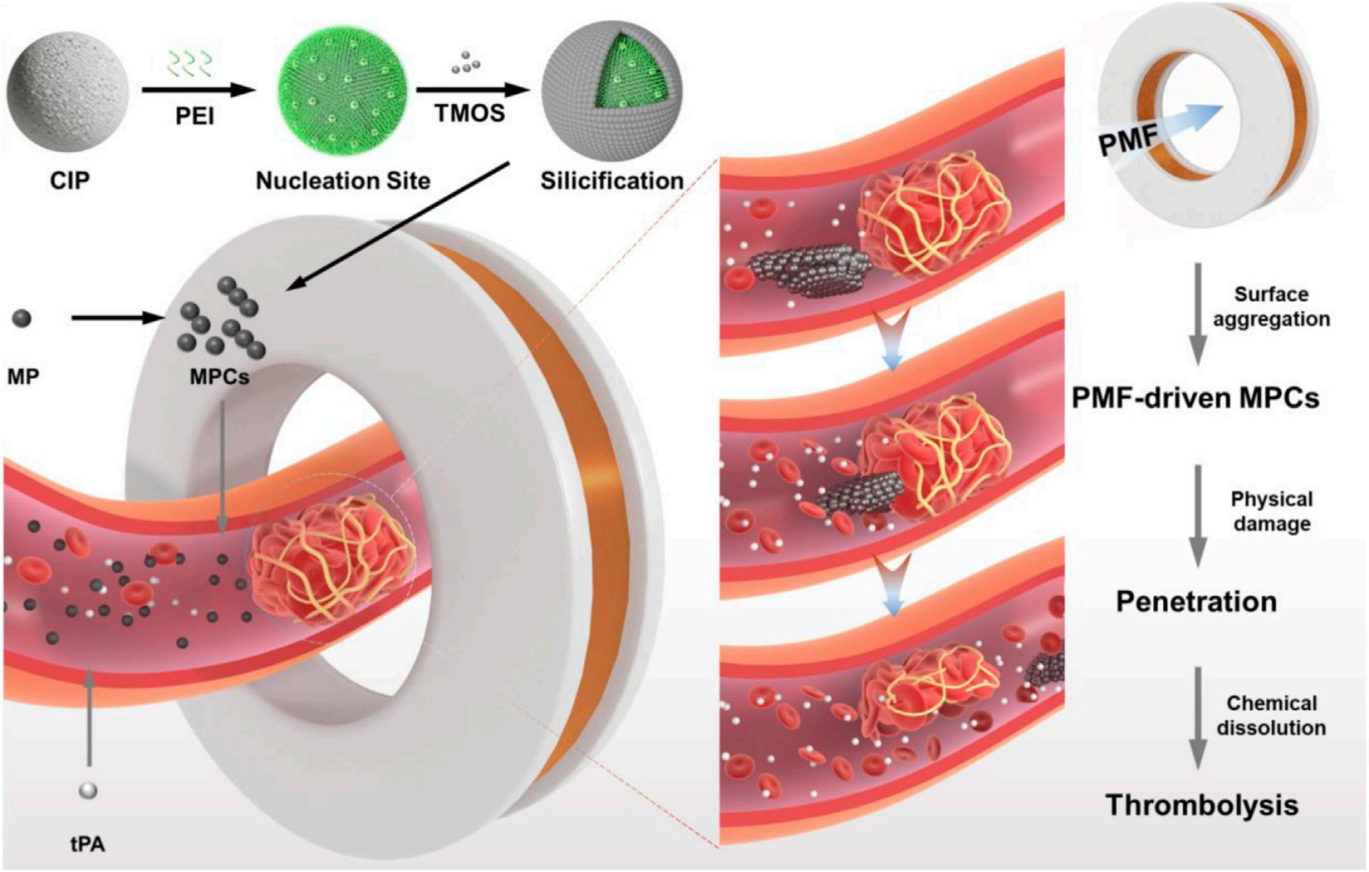
Schematic of the thrombolysis technique using PMF-driven MPCs. After MPCs mixed with tPA are aggregated near the blood clot, a PMF is applied through an electromagnetic coil to guide the clusters in penetrating the clot, forming a through-channel which enhances the thrombolytic efficacy of the fibrinolytic enzyme.

In summary, existing magnetically controlled thrombolysis studies have made substantial progress in magnetic swarm structural design and external field configuration optimization. However, the introduction of relatively complex micro/nano structures and multi-modal external excitation has increased the technical implementation cost and complexity. Furthermore, the prevailing approach of conventional magnetically controlled thrombolysis primarily relies on the principle of magnetic torque-induced friction, thus rendering MPCs unable to rapidly penetrate thrombi within 10 min. Therefore, the rapid development of convenient and reliable means to break through the thrombus structural barrier is a meaningful and critical issue. A pulsed magnetic field (PMF) is a transient field characterized by a short duration and a high amplitude (Lei et al., 2025). Based on the regulatory characteristics of PMF on magnetic materials (Zablotskii et al., 2011; Camacho and de Vicente, 2025), the present study pioneers the establishment of a targeted thrombolysis technology that utilizes PMF to drive MPCs. By adjusting PMF parameters, MPCs can be driven to physically disrupt the thrombus structure within minutes and accelerate drug-mediated chemical thrombolysis, as outlined in Figure 1. This provides new insights for developing novel and efficient magnetically controlled thrombolysis technologies.

## 2 Materials and methods

### 2.1 Fabrication of CIP@SiO_2_ particles

**FIGURE 2:**
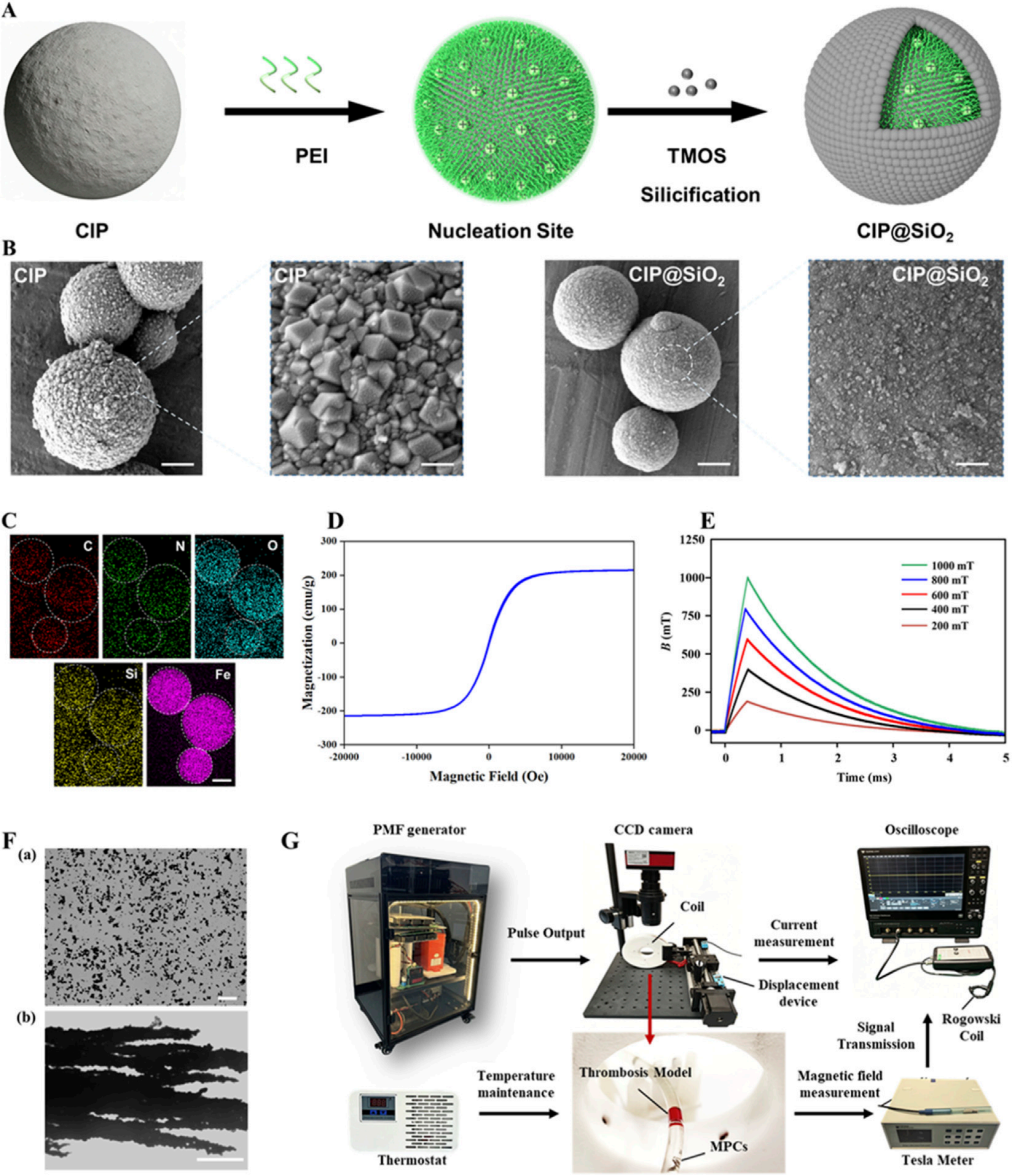
The pulsed magnetically controlled thrombolysis platform. **(A)** Schematic diagram of the CIP@SiO_2_ synthesis process. **(B)** Overall and local morphological features of CIP and CIP@SiO_2_ under scanning electron microscopy. The scale bar is 1 μm in the overall image and 200 nm in the local image. **(C)** Elemental mapping of CIP@SiO_2_ with detected element signals distributed across the MPs. Scale bar = 1 μm. **(D)** Hysteresis loop of the CIP@SiO_2_. **(E)** Measured PMF waveform along the central axis at a distance of 15 mm from the coil center. **(F)** Morphological distribution of MPCs: (a) Without an applied magnetic field, (b) Under PMF. Scale bar = 50 μm. **(G)** Overall display of the pulsed magnetically controlled thrombolysis experimental platform.

To enable rapid response to PMF, carbonyl iron powder (CIP) was selected as the magnetic particle substrate due to its high purity and good biosafety. To further improve the biocompatibility and colloidal stability of CIP, surface silicification modification was performed, as illustrated in Figure 2A.

Specifically, to introduce positive charges on the CIP surface, 100 mg of CIP was gradually added to 25 mL of deionized water containing 25 mg of polyethyleneimine (PEI) under vigorous stirring, to prepare a positively charged CIP dispersion. Subsequently, a separate silicification precursor solution was prepared by dissolving 750 µL of tetramethylorthosilicate (TMOS) in an acetic acid buffer (pH 3.0) under vigorous stirring. The two solutions were then mixed and reacted on a vortex mixer for 24 h, yielding a silicified CIP dispersion, where the final concentration of silicon species was 128 mmol/L.

The product was purified by repeated centrifugation (8000 rpm, 10 min) to remove the supernatant, followed by magnetic separation to collect the high-purity silicified CIP particles (CIP@SiO_2_).

### 2.2 Structural, morphological, and dispersion characterization

The morphology and surface structure of CIP and CIP@SiO_2_ particles were characterized using a field-emission scanning electron microscope (GeminiSEM 300, ZEISS, Germany). SEM images were acquired at an electron high tension (EHT) of 3 kV. The elemental distribution of CIP@SiO_2_ particles was analyzed by energy-dispersive X-ray spectroscopy (EDS) mapping using the EDS detector equipped on the SEM system. EDS mapping was performed at an EHT of 15 kV.

The particle size distribution of CIP@SiO_2_ was determined from SEM images using ImageJ. A total of 5 randomly selected SEM fields were analyzed, corresponding to an analyzed area of approximately 13,500 μm^2^. At least 200 individual particles were measured using ImageJ. The particle diameter was calculated as the equivalent circular diameter.

The aggregation morphology of CIP@SiO_2_ particles under PMF actuation was further evaluated using optical microscopy. Briefly, 0.2 mg of CIP@SiO_2_ particles was dispersed in 100 μL of PBS and transferred into a PVC tube. The particle suspension was observed before and after PMF auction to record the PMF-induced aggregation morphology of MPCs using an optical microscope (MATEO TL RUO, Leica, Germany). In addition, the initial distribution of CIP@SiO_2_ particles in whole blood without magnetic-field stimulation was assessed to evaluate their spontaneous aggregation behavior in a blood environment. Fresh whole blood (1 mL) was spread in a 35 mm Petri dish, followed by the addition of 0.2 mg of CIP@SiO_2_ particles. The sample was then placed under an optical microscope to observe the no-field distribution state of the particles in whole blood.

### 2.3 Preparation of thrombus model in vitro

Porcine whole blood was used to prepare *in vitro* thrombus models, as porcine blood is widely recognized as an appropriate surrogate for human blood in coagulation studies, and the resulting clots exhibit histological characteristics comparable to those of human thrombi. Fresh porcine blood was purchased from Zhengzhou Pingrui Biotechnology Co., Ltd. (Zhengzhou, China) and mixed with an anticoagulant solution (citrate–glucose solution, C3821, Sigma-Aldrich, Singapore) at a volume ratio of 9:1. The anticoagulated blood was stored at 4 °C and used within 2–3 weeks.

To prepare red thrombi, a 0.5 M CaCl_2_ solution was added to fresh porcine whole blood at a volume ratio of 1:50 and mixed thoroughly to initiate coagulation. A predefined volume of the blood mixture was then rapidly injected into custom molds using a microsyringe, with the entire transfer process completed within 5 min to prevent premature clotting inside the syringe. The molds containing the blood samples were subsequently incubated in a 37 °C incubator (Thermo Fisher Scientific, United States) for at least 12 h to allow the formation of stable red thrombi.

For the preparation of white thrombi, fresh porcine whole blood was first centrifuged at 120 *g* for 20 min at room temperature to obtain platelet-rich plasma (PRP). The supernatant was carefully collected, and CaCl_2_ solution was added to the PRP or fluorescently labeled plasma to achieve a final CaCl_2_ concentration of 25 mM, followed by gentle mixing to trigger coagulation. The plasma mixture was immediately injected into molds using a microsyringe to ensure complete transfer prior to clot formation. The molds were then incubated at 37 °C for at least 12 h, resulting in the formation of contracted white thrombi.

### 2.4 Thrombolytic performance test

*In vitro* thrombolysis experiments were conducted using polyvinyl chloride (PVC) tubes as the experimental vessels. Throughout the experiments, the temperature of the thrombi in both the experimental and control groups was strictly maintained at the same level to ensure experimental consistency.

#### 2.4.1 Penetrating thrombus experiment

The experiments were performed in a static-flow tube model. No external perfusion pump or imposed background flow was applied during treatment, and the initial bulk-flow velocity around the thrombus was set to zero. Before magnetic actuation, 100 μL of normal saline was introduced at each end of the thrombus sample to maintain a hydrated local environment. MPCs at the predetermined dose were then introduced from one end of the tube to mimic proximal catheter-based particle delivery. During PMF treatment, the central axis of the excitation coil was aligned with the longitudinal axis of the thrombus model to ensure directional consistency between the magnetic field and the target thrombus region. The number of applied PMF pulses was recorded throughout the experiment.

To record the dynamic penetration process, the thrombus model and MPCs were monitored in real time using a CCD camera (4K 200D, DEYI). For red thrombi, penetration was considered complete when MPCs were observed exiting the thrombus from the distal side. The corresponding pulse number at this point was recorded, and the average penetration length per single pulse (APL-SP) was calculated as the ratio of thrombus length to pulse number.

For white thrombi, because of their partial light transmittance, the penetration front of MPCs within the thrombus was directly recorded. The penetration distance was measured from calibrated images using ImageJ. The APL-SP was determined by dividing the measured penetration distance by the corresponding pulse number.

#### 2.4.2 *In Vitro* thrombolysis experiment

To investigate the effect of PMF-driven MPCs-mediated penetration through thrombi on subsequent tPA-mediated thrombolysis, thrombi penetrated by MPCs were designated as the experimental group, while intact thrombi served as the control group. Prior to the experiment, 100 μL of normal saline was injected into one side of each sample, and 100 μL of tPA solution (25 μg/mL) was added to the other side. For the experimental group, 0.2 mg of MPCs was additionally mixed into the tPA solution, followed by PMF-driven penetration with a preset number of penetrations immediately after the experiment initiation.

The thrombolysis rate was quantified by measuring the thrombus mass loss before and after PMF treatment. Prior to clot formation, the mass of the empty mold was measured using a high-precision electronic balance (FA124C, LIHEN) and defined as *m*_c0_. Before the application of any treatment, the combined mass of the mold containing the intact thrombus was measured and defined as *m*_c1_. After PMF exposure, the flowing mixed solution and MPCs inside the mold were carefully removed. During this process, the collected solution was filtered using filter paper to examine whether any detached thrombus fragments were present. Subsequently, the combined mass of the mold containing the remaining thrombus was measured again and defined as *m*_c2_. Based on the measured mass values, the mass-based thrombolysis rate *v*_lysis_ was calculated according to Equation 1:

$$v_{\text{lysis}\;}=\frac{m_{c1}-m_{c2}}{\left(m_{c1}-m_{c0}\right)t_{m}}$$ (1)

Where, *t*_m_ denotes the thrombolysis duration during experimental observation.

#### 2.4.3 Residual MPs evaluation after thrombus penetration

Residual MPs after thrombus penetration and magnetic retrieval were evaluated using a CCD camera and an optical microscope. The penetration cycle number was set to 15, and the total mass of MPs used to form MPCs was set to 0.2 mg. After MPCs penetrated the thrombus, a neodymium–iron–boron (NdFeB) permanent magnet was immediately used to retrieve the MPs and separate them from the tube. The liquid released around the thrombus after MPCs penetration was collected immediately and transferred onto a glass slide for microscopic observation. After complete thrombus dissolution, the remaining lysate in the tube was also collected and observed under the same imaging conditions. The presence or absence of residual MPs in the collected samples was recorded based on microscopic images.

#### 2.4.4 Dynamic-flow thrombolysis assay after PMF-driven MPCs penetration

A dynamic-flow thrombolysis experiment was performed to further evaluate the performance of PMF-driven MPCs under continuous perfusion. Thrombi were placed in a PVC tube with an inner diameter of 2 mm. A syringe pump (SHP05, SH) was used to continuously perfuse tPA solution (20 μg/mL) through the tube at a volumetric flow rate of 10 mL/hr. This flow condition was selected to provide a controlled low-flow environment relevant to thrombolytic infusion and small-vessel hemodynamics (Khalil et al., 2017). MPCs were introduced into the tube under continuous tPA perfusion and actuated by the PMF system to penetrate the thrombus. After penetration was completed, a permanent magnet was used to retrieve the MPs from the tube. The subsequent thrombus dissolution process under continuous flow was monitored using a CCD camera until complete thrombus dissolution.

### 2.5 Biosafety test of CIP@SiO_2_

#### 2.5.1 Cell culture and viability assay

NIH/3T3 cells and human umbilical vein endothelial cells (HUVECs) were obtained from the Biomedical Analysis Center, Army Medical University. NIH/3T3 cells were cultured in DMEM, whereas HUVECs were cultured in RPMI 1640 medium. Both media were supplemented with 10% fetal bovine serum (Life Technologies) and 1% penicillin/streptomycin (Capricorn) at 37 °C in a humidified atmosphere containing 5% CO_2_.

For the cell viability assay, NIH/3T3 cells and HUVECs were seeded respectively into 96-well plates at a density of 5 × 10^4^ cells mL^-1^ (100 μL per well). After 12 h to allow cell attachment, the culture medium was replaced with 100 μL of fresh complete medium containing CIP or CIP@SiO_2_ particles at different concentrations, with the highest concentration set at 10 mg/mL. Cells in the control group received particle-free complete medium. After 24 h of co-culture, the treatment medium was removed and replaced with fresh complete medium containing 10% CCK-8 reagent, followed by incubation for an additional 2 h. The absorbance at 450 nm was measured using a Multiskan Go microplate reader to evaluate cell viability.

#### 2.5.2 Hemolysis assay under PMF treatment

A hemolysis assay was performed to evaluate whether CIP@SiO_2_ particles induced red blood cell rupture under PMF treatment. Briefly, 2 mL of fresh anticoagulated porcine whole blood was mixed with 5 mL PBS and centrifuged at 1500 rpm for 10 min to collect red blood cells. After removing the supernatant, the red blood cells were washed three times with PBS and resuspended in 10 mL PBS to prepare the red blood cell suspension. Deionized water and PBS were used as the positive and negative controls, respectively. CIP@SiO_2_ particles at different concentrations were dispersed in PBS to prepare 1 mL particle suspensions. Then, 1 mL of particle suspension or control solution was mixed with 1 mL of red blood cell suspension and treated with PMF at 1 T and 1 Hz for 5 min, followed by incubation at 37 °C for 3 h. After incubation, the samples were centrifuged at 1500 rpm for 5 min, and the absorbance of the supernatant at 540 nm was measured using a microplate reader (BioTek Epoch 2, Agilent, United States). The hemolysis rate *R*_he_ was calculated according to Equation 2:

$$R_{\text{he}}=\frac{A_{m}-A_{n}}{A_{p}-A_{n}}\times 100\%$$ (2)

Where *A*_m_ is the absorbance of the supernatant from the particle-treated group, *A*_n_ is the absorbance of the PBS negative control group, and *A*_p_ is the absorbance of the deionized water positive control group.

### 2.6 Statistical analysis

All data were analyzed using GraphPad Prism 9 (GraphPad Software, Inc., San Diego, CA, United States) and MATLAB 2024b (MathWorks, Inc., Natick, MA, United States). All experiments were repeated three times and the number of samples in each group was 3–6. Data were first tested for normality (Shapiro-Wilk test) and homogeneity of variance (Levene’s test). Results are presented as mean ± standard deviation (SD) unless otherwise stated. Accordingly, one-way ANOVA followed by Dunnett’s *post hoc* test was used for multiple comparisons between the control group and each treatment group. Statistical significance was set at P < 0.05, with significance levels denoted as *P < 0.05, **P < 0.01, and ***P < 0.001.

### 2.7 PMF-driven MPCs thrombolysis platform

The present study used a tube with an inner diameter of 2 mm to construct the *in vitro* thrombus model, which is within the millimeter-scale range commonly used in representative *in vitro* thrombus or vascular models (Wang et al., 2021; 2024; Tang et al., 2022). To satisfy the requirements of both *in vitro* thrombolysis and animal experimentation, a multi-turn solenoid coil with an inner diameter of 4 cm was designed. This inner diameter provides sufficient radial clearance for the 2 mm PVC tube, sample holder, and optical observation during *in vitro* experiments. Meanwhile, it is also compatible with the local hindlimb or femoral-vessel region of mice or SD rats in small-animal thrombus experiments, while leaving additional space for animal positioning and operation (Yang et al., 2023; Wu et al., 2024). The electromagnetic coil was manually wound using 1.2 mm-diameter polyimide-insulated copper wire and consisted of eight layers with 35 turns in each layer. Therefore, this multi-turn solenoid configuration was selected to provide sufficient PMF output over the target region while maintaining structural compatibility with both *in vitro* and small-animal thrombolysis experiments.

To enable flexible adjustment of the coil position and actuation direction, a single-axis translation stage was employed to control the electromagnetic coil. By regulating the magnetic force generated by the coil, the motion of MPCs was effectively confined within the thrombus region. The PMF generator used in this study incorporated a circuit module from a previously developed modular, energy-efficient system (Lei et al., 2024), with the pulsed magnetic field generated through capacitor discharge. To maintain thermal stability during the experiments, a temperature control unit was used to ensure that the thrombus model remained at room temperature throughout the testing process. Real-time imaging of the thrombus model and MPCs dynamics was performed using a CCD camera (4K 200D, DEYI), which provides 4K resolution and a frame rate of up to 200 frames per second. This setup enabled clear visualization of the morphological evolution of MPCs as well as the thrombolysis process.

The electrical and magnetic characteristics of the system were monitored using multiple measurement instruments. A Rogowski coil (CWTMini, HF6B) was employed to measure the current waveform of the electromagnetic coil, while a T m (Model 1600, CH-Hall) was used to quantify the magnetic field output. The temperature variation of the PMF device was monitored in real time using a thermocouple thermometer (TA612C, TRSI). All signals were recorded and visualized using an oscilloscope (HDO6054B, Teledyne LeCroy), enabling accurate and real-time monitoring of both circuit operation and magnetic field characteristics. Finally, the constructed pulsed magnetically controlled thrombolysis platform is shown in Figure 2G.

## 3 Results

### 3.1 Characterization of CIP@SiO_2_ magnetic particles

In order to respond rapidly to PMF, the present study employed carbonyl iron powder (CIP), which exhibits high purity and good biosafety, as the magnetic particle substrate. To further enhance the biocompatibility of the MPs, surface silicification (CIP@SiO_2_) was performed on the CIP as illustrated in Figure 2A. The surface morphology of the MPs before and after silicification was characterized by scanning electron microscopy (Figure 2B). The results indicate that compared with unmodified CIP, the CIP@SiO_2_ particles exhibit a smoother surface and a predominantly spherical morphology. Detailed particle size distributions are presented in Supplementary Figure S1 with an average particle diameter of 3.27 µm. At higher magnification, the presence of a nanoscale silicon dioxide crystalline layer coating the surface of the CIP@SiO_2_ particles was observed.

As illustrated in Figure 2C, the elemental composition of CIP@SiO_2_ particles shows obvious silicon and oxygen signals on the particle surface. Supplementary Figure S2 presents the elemental mapping results of CIP@SiO_2_ particles, and the corresponding quantitative EDS elemental composition is summarized in Supplementary Table S1. The low Si content suggests that SiO_2_ was mainly localized as a thin surface layer on the micron-sized Fe-rich CIP core, supporting the formation of a core–shell-like surface structure. In addition, the peaks observed at approximately 8–9 keV were assigned to Cu Kα and Cu Kβ signals from the copper foil substrate used for sample loading, rather than from the CIP@SiO_2_ particles themselves. Together with the elemental mapping results, these findings support the successful silicification of CIP particles. Figure 2D presents the magnetic hysteresis curve of CIP@SiO_2_ particles. The saturation magnetization of the samples reached approximately 210 emu/g, with a negligible remanence, thus demonstrating excellent magnetic responsiveness that is suitable for the requirements of this study. The macroscopic aggregation morphology of MPCs under PMF actuation was observed and recorded using an optical microscope. Figures 2F a, b show the morphological distribution of MPCs under no-field and pulsed magnetic field-driven conditions, respectively. Without an applied field, the particles are dispersed. Under PMF actuation, magnetic attraction between particles leads to the formation of compact, needle-like aggregates exceeding several hundred micrometers in size, as shown in Supplementary Figure S3. These elongated aggregates provided a favorable structural basis for localized interaction with the thrombus surface and subsequent thrombus disruption. The no-field distribution of CIP@SiO_2_ particles in whole blood was further examined to assess their spontaneous clustering behavior. As shown in Supplementary Figure S7, CIP@SiO_2_ particles exhibited a relatively uniform distribution in whole blood without PMF actuation, and no obvious spontaneous aggregation was observed. This result suggests that CIP@SiO_2_ particles did not intrinsically form large clusters in a blood-containi ng environment under no-field conditions.

The cytocompatibility of CIP and CIP@SiO_2_ particles was evaluated using NIH/3T3 cells and HUVECs (Supplementary Figure S4). After 24 h of co-culture with CIP@SiO_2_ particles, both NIH/3T3 cells and HUVECs maintained high viability even at the highest tested concentration of 10 mg/mL, and no significant decrease was observed compared with the particle-free control group (Supplementary Figures S4b,d). In contrast, co-culture with unmodified CIP particles resulted in a concentration-dependent decrease in cell viability in both cell types (Supplementary Figures S4a,c). These results indicate that SiO_2_ modification effectively improved the cytocompatibility of the magnetic particles and reduced the particle-induced inhibitory effect on cell viability, which is consistent with the findings of previous studies (Turcheniuk et al., 2013; Boustani et al., 2018). The hemolysis results in Supplementary Figure S6 showed that, within the tested concentration range of 0–5000 μg/mL, all groups exhibited hemolysis rates of approximately 2%–3%, which were below the 5% threshold commonly used for biomaterial hemocompatibility evaluation (Malehmir et al., 2023). No significant difference was observed between the particle-treated groups and the PBS negative control group. These results indicate that CIP@SiO_2_ particles did not induce obvious red blood cell rupture even at concentrations higher than those used in the thrombus penetration experiments.

### 3.2 Generation and thermal characterization of exponential PMF waveforms

The exponential waveform is a frequently employed PMF waveform in the domain of electromagnetic pulse regulation, a consequence of its considerable adjustability and ease of implementation. When the electromagnetic coil is supplied with an exponential pulsed current, a corresponding PMF waveform is obtained. The amplitude of the PMF is a key parameter for in the control of the collective motion of MPCs. It has been demonstrated that when driven by exponential current pulses with peak values ranging from 50 A to 250 A, measured results demonstrate that the coil generates exponential PMF with peak values ranging from 0.2 T to 1 T along its axis at a distance of 15 mm from the center. The specific magnetic field waveforms are illustrated in Figure 2E. While the coil temperature remains within an acceptable range of below 40 °C, the frequency of the PMF can be flexibly adjusted from 0.05 Hz to 1 Hz, thus meeting the experimental requirements for parameter control.

At the maximum pulse frequency used in this study (1 Hz), the temperature at the inner wall of the coil was recorded for 5 min under different PMF amplitudes. As shown in Supplementary Figure S5, the coil temperature increased gradually during continuous PMF output. However, the temperature remained below 40 °C under all tested pulse amplitudes, and the temperature difference between the lowest and highest PMF amplitudes did not exceed 5 °C after 5min of operation. Because the experimental target region was separated from the coil by an air gap, the effect of coil heating on the local thrombus environment was considered negligible.

### 3.3 Simulation and analysis of electromagnetic force characteristics

**FIGURE 3:**
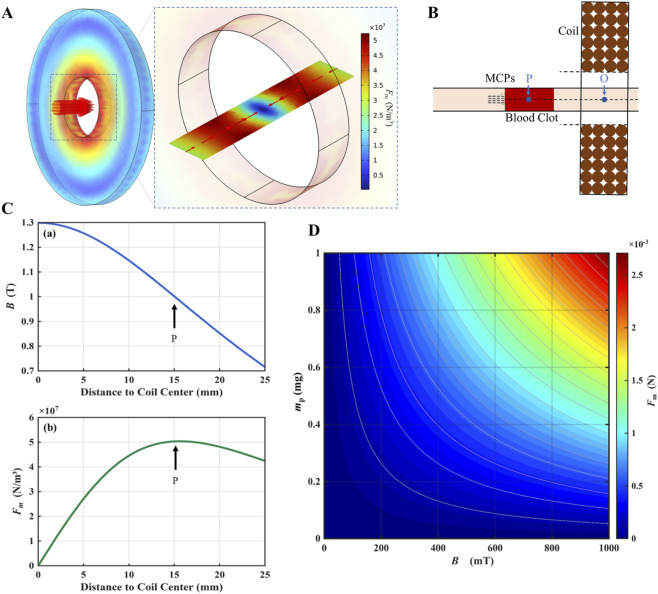
Simulation of electromagnetic force and establishment of pulse action scheme. **(A)** Spatial Electromagnetic Force Distribution of the Electromagnetic Coil. **(B)** Relative Position of the Electromagnetic Coil and Thrombus Vessel in the Pulsed Magnetic Control Scheme. **(C)** Axial variations in electromagnetic force magnitude and magnetic field strength from the coil center: (a) electromagnetic force amplitude, (b) magnetic field intensity. **(D)** Two-dimensional contour plot showing the dependence of 0.2 mg MPCs on magnetic field strength *B* and magnetic particle quality *m*_p_.

To analyze the electromagnetic force distribution generated by the coil, a full-scale coil model was established via the finite element analysis (FEA) software Comsol Multiphysics (Figure 3A). When a 250 A current is applied to the coil model, the simulation results show that the direction of the electromagnetic force within the coil’s axial region is parallel to the axis and points toward the coil center. Figure 3C illustrates the variations in magnetic field strength and magnetic force along the coil’s central axis relative to the distance from the coil center. Specifically, Figure 3Ca indicates that the axial magnetic field strength decreases as the distance from the coil center increases, while Figure 3Cb shows that the axial electromagnetic force first increases along the direction toward the coil and then rapidly decays inside the coil. Therefore, by adjusting the axial orientation of the coil, the direction of motion of MPCs within the axial region can be flexibly controlled. For MPCs moving outside the coil, they are confined to the coil’s central region under the traction of the pulsed magnetic force, ensuring the safety and reliability of the magnetic control process. The relative configuration between the electromagnetic coil and the thrombus in the pipe is shown in Figure 3B, with the central axis of the coil aligned with the geometric centerline of the thrombus. Point P is defined as the geometric center of the thrombus and serves as the reference location for magnetic field measurement. According to Figure 3C, point P is positioned at a distance of 15 mm from the coil center. In this region, the magnetic force reaches its peak and exhibits a relatively smooth variation.

Both the magnetic flux density *B* and particles mass *m*_p_ affect the force magnitude on MPCs (Zhou et al., 2016). Considering interfering factors such as MPCs magnetization saturation and combining the established coil model, we focused on the total magnetic force acting on MPCs at point P. Subsequently, MATLAB was employed to plot the two-dimensional distribution characteristics of the force as a function of the applied *B* and *m*_p_, as illustrated in Figure 3D. Color gradients characterize the variation in force magnitude, with white curves representing isoforce lines. The results indicate that within the commonly used MPCs mass range in research, applying a magnetic field of 0-1000 mT can generate an electromagnetic force on MPCs at the millinewton (mN) level, with a maximum value of 2.5 mN. This force magnitude exceeds the reported yield strength of fibrin-dominated thrombi, suggesting that PMF-driven MPCs are theoretically capable of mechanically disrupting the clot structure (Münster et al., 2013). Furthermore, the inclined distribution of isoforce lines reflects a synergistic effect between the variables. Specifically, to maintain the same force magnitude, lower magnetic field intensities require matching with larger particle masses, while smaller particles necessitate higher magnetic field intensities for compensation. Thus, the magnetic force on MPCs exhibits a positive correlation with both *B* and *m*_*p*_, and the two parameters exhibit regulatory coupling. The deficiency of a single parameter can be compensated by increasing the other to achieve the same force level.

### 3.4 Investigation on thrombus penetration behavior of PMF-driven MPCs

#### 3.4.1 Study on penetration efficiency of PMF-driven MPCs

**FIGURE 4:**
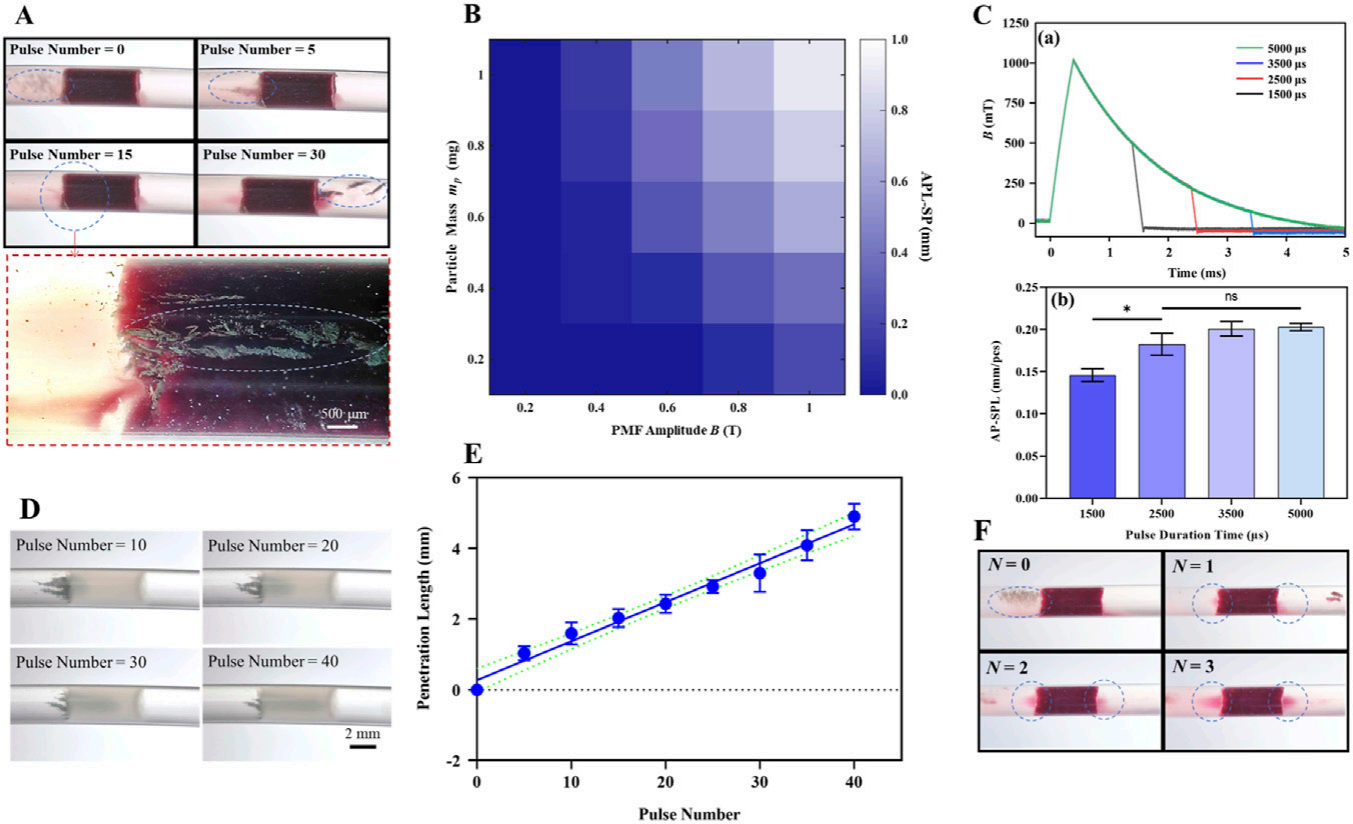
Study on Penetration Efficiency of PMF-driven MPCs. **(A)** Penetration process of PMF-driven MPCs through red thrombi, with the blue dashed ellipse in the image illustrating the changes in MPC morphology under pulsed drive and during the penetration process. Scale bar = 500 μm. **(B)** The influence of magnetic flux density *B* and particle mass *m*_p_ on APL-SP (*n* = 6). **(C)** Optimization of penetration waveform for PMF-driven MPCs: (a) Pulsed tail-truncated waveform, (b) Relationship between APL-SP and pulsed tail duration under tail-truncated waveform conditions (*n* = 3). **(D)** Images of the MPCs penetrating through white thrombi. Scale bar = 2 mm. **(E)** Relationship between pulse number and penetration length (*n* = 5). **(F)** Multiple penetration processes of PMF-driven MPCs, with the blue dashed circle in the image indicating detached RBCs during reciprocating penetration.

To demonstrate the regulatory effect of the PMF, *m*_p_ = 0.2 mg and *B* = 1 T were set. Figure 4A illustrates the process of MPCs gradually penetrating the blood clot under the guidance of the PMF. Before the start of penetration (i.e., Pulse Number = 0 pcs), MPCs exhibit a dispersed distribution inside the vessel. With the continuous accumulation of applied PMF counts, MPCs can be observed transforming from a dispersed state to an aggregated state and accumulating at the thrombus interface. Subsequently, MPCs penetrate into the interior of the blood clot. When Pulse Number = 30 pcs, MPCs pass through the blood clot from the inside out, indicating that the blood clot has been unidirectionally penetrated. At a pulse frequency of 0.05 Hz, the entire unidirectional penetration process required only 10 min. Increasing the pulse frequency to 1 Hz shortens the penetration time to 30 s.

Since increasing the magnetic particle dosage raises biosafety risks, it cannot be arbitrarily increased. While enhancing the PMF intensity not only escalates the power requirement of the generator but also causes higher and more uncontrollable coil temperatures, thereby compromising the safety of the operating environment. Therefore, neither the *B*, nor *m*_*p*_ can be increased indefinitely. The optimal solution is to select parameters with the highest efficiency within a reasonable range. To calibrate and normalize the average penetration efficiency corresponding to different combinations of *B* and *m*_*p*_, a red thrombus of approximately 6 mm in length was set as the total penetration distance. By recording the penetration completion time and the total number of pulses consumed in each group of experiments, the APL-SP of MPCs through the red thrombus under the influence of different variables was calculated. Figure 4B illustrates the coupled regulatory law of *B* and *m*_*p*_ on thrombus penetration efficiency. The color gradient in the heatmap intuitively reflects the spatial distribution characteristics of penetration velocity, revealing the synergistic mechanism between external PMF and particle parameters. Under the condition of fixed particle mass, the APL-SP shows a significant increasing trend with the enhancement of PMF intensity. For instance, when the particles mass is 0.6 mg, as the PMF increases from 0.2 T to 1 T, the corresponding APL-SP jumps from 0 to approximately 0.64 mm. This phenomenon stems from the direct enhancement effect of magnetic flux density on the driving force. When the PMF intensity is fixed, the APL-SP increases with the rise of *m*_*p*_. For example, at *B =* 0.6 T, as *m*_*p*_ increases from 0.2 mg to 1.0 mg, the corresponding APL-SP rises from 0 mm to 0.41 mm, which is attributed to the magnetic moment accumulation effect of particle clusters. The continuous color gradient along the diagonal direction of the heatmap reveals the synergistic optimization path of PMF and mass: in the region where *B* > 0.6 T and *m*_p_ > 0.6 mg, the corresponding APL-SP values are all greater than 0.3 mm, indicating that the magnetic force is significantly larger than the thrombus resistance at this point. Notably, APL-SP exhibits an obvious threshold effect in the 0.2 T low PMF region: the yield stress of the thrombus matrix requires a minimum magnetic force to overcome, with this critical value representing the penetration initiation boundary.

#### 3.4.2 Optimization of penetration waveform for PMF-driven MPCs

Traditional exponential wave PMF undergo exponential decay after reaching the peak magnetic field amplitude to form a long pulse width. This decay process of magnetic field intensity is accompanied by increased coil heat and energy consumption. However, low-amplitude magnetic fields cannot provide sufficient penetration. Therefore, pulse width regulation is necessary to enhance energy utilization efficiency. The exponential wave pulse width is modified by truncating the pulsed tail duration, as shown in Figure 4Ca. To investigate the influence of different pulsed tail durations on penetration velocity, the APL-SP of MPCs with *m*_p_ = 0.2 mg under pulses of varying widths was recorded. Figure 4Cb indicates that when the pulsed tail duration exceeds 3500 μs, further increasing the pulse duration no longer significantly improves APL-SP. This result further reveals that when the PMF intensity decreases to approximately 200 mT, the magnetic force driving MPCs is no longer sufficient to achieve significant thrombus penetration. Therefore, adjusting the exponential wave pulsed tail duration to the range of 2500-3500 μs constitutes an optimal magnetic control scheme.

#### 3.4.3 Dynamic response of PMF-driven MPCs during white blood clot penetration

To further analyze the time-varying law of MPCs-mediated penetration through the thrombus during pulse application, the penetration length of MPCs was real-time recorded as the number of pulses increased. Due to the certain light transmittance of white blood clots, it is possible to intuitively visualize whether the MPCs have penetrated into the thrombus and their penetration length. Therefore, a 6 mm-long white thrombus was selected as the experimental target. A CCD camera was employed to monitor the MPC-mediated penetration process within the thrombus in real time after the application of intermittent PMF (Figure 4D). The variation law of MPC-mediated penetration length with the number of pulses applied, as illustrated in Figure 4E. Experimental results indicate that as the number of pulses applied increases, the penetration length of MPCs exhibits a nearly continuous linear growth trend. Owing to the relatively uniform magnetic force in the actuation region, the penetration length corresponding to the same number of pulses demonstrates consistency, indicating that precise control of penetration length can be achieved by regulating the pulse count.

#### 3.4.4 Multiple penetration of PMF-driven MPCs

Since MPCs have already formed a penetrating channel after the first thrombus penetration, when the coil applies electromagnetic force in the opposite direction, MPCs can quickly traverse the thrombus along the original channel, achieving further penetration of the thrombus. To intuitively demonstrate this process, this study set the operating parameters as *m*_p_ = 0.2 mg and *B* = 1 T, and conducted multiple penetration tests. Figure 4F illustrates the changes in the surrounding solution environment of the red thrombus before MPCs action (penetration number *N* = 0), after single penetration, and during multiple penetration processes. It can be observed that when MPCs first penetrate to the opposite side of the thrombus from the initial position, they induce the detachment of red blood cells (RBCs) from the thrombus. As the number of reciprocating penetrations by MPCs increases, the red area overflowing from the thrombus sides expands and darkens, indicating more RBCs detachment during penetration.

### 3.5 Penetration-enhanced thrombolytic performance and post-treatment evaluation of PMF-driven MPCs

#### 3.5.1 Thrombolysis enhancement via penetration of PMF- driven MPCs

**FIGURE 5:**
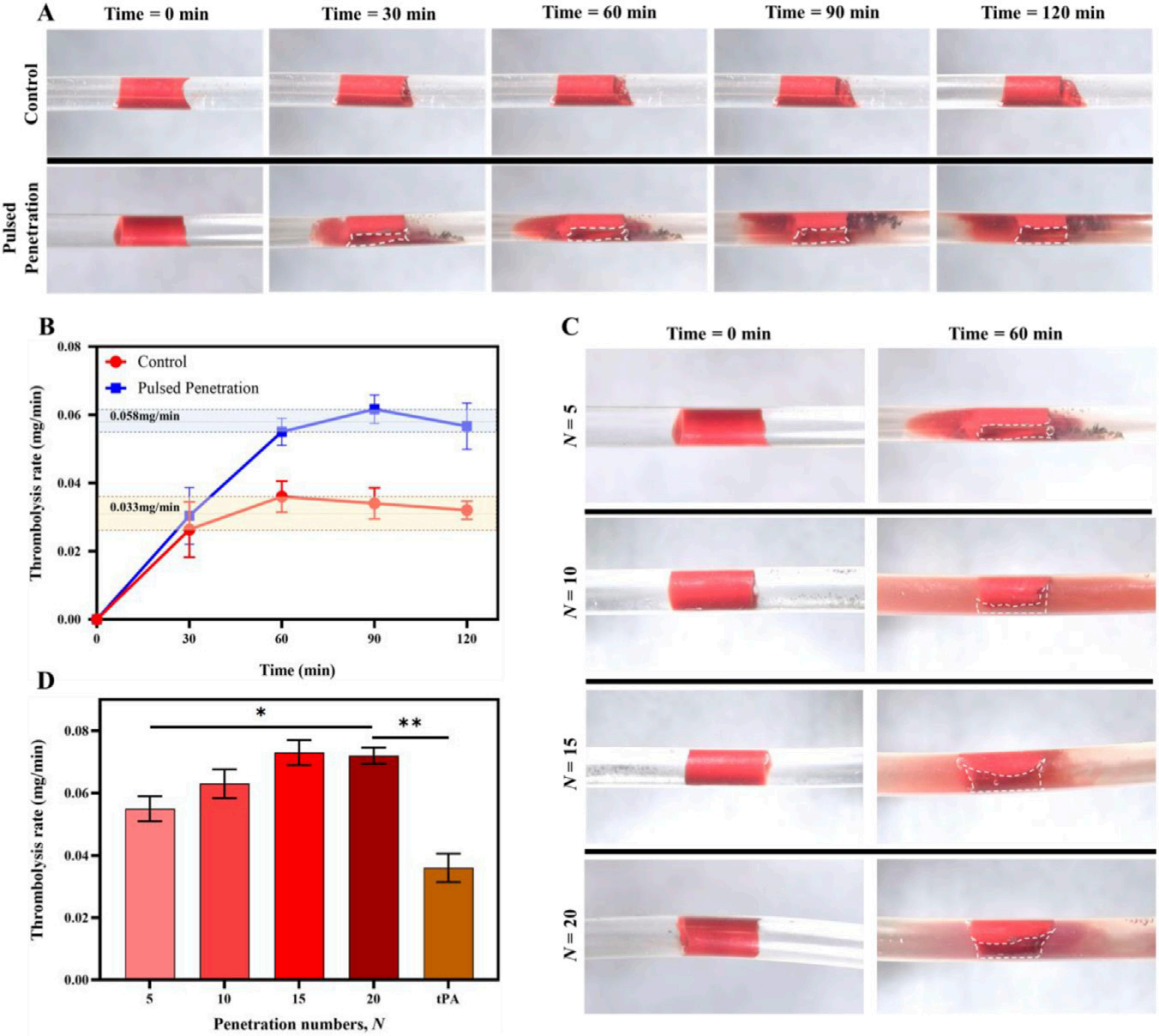
**(A)** Time-dependent changes in blood clot status for the pulsed penetration thrombolysis experimental group and the tPA-only control group, the white dashed lines in the figure outline the contour of the through passage. **(B)** Thrombolytic rate curves of the pulsed penetration thrombolysis experimental group and the tPA-only control group (*n* = 5). **(C)** Changes in blood clot status changes in the pulsed penetration thrombolysis experimental group before and after 60 min under different pulsed penetration numbers *N*. **(D)** Comparison of thrombolytic efficiency in the pulsed-penetration thrombolysis experimental group under different penetration numbers *N* (*n* = 3).

To investigate the effect of PMF-driven MPC-mediated penetration through thrombi on subsequent tPA-mediated thrombolysis, Figure 5A illustrates the morphological changes of thrombi in each group at different time points within 120 min. At 30 min post-experiment initiation, a narrow penetrating channel (marked by white dashed lines) spanning both ends of the blood clot was observed in the experimental group. With the formation of the channel, partial substances precipitated from the exposed side of tPA. At this stage, the mass dissolution rate of the blood clot reached approximately 0.03 mg/min, slightly higher than that of the control group but without significant difference (Figure 5B). This indicates that the thrombolytic effect in both groups during this period was mainly attributed to the unilateral interface contact reaction between the drug and the blood clot. Furthermore, the experimental group displayed accelerated channel expansion, accompanied by continuous leakage of partially dissolved thrombus material, which indicated complete tPA penetration into the clot channel and enhanced thrombolytic activity. In contrast, the control group treated with tPA alone showed only localized dissolution confined to one side of the thrombus. During the 30–60 min period, the thrombolytic rate of the experimental group increased rapidly, while that of the control group reached saturation. This significant difference demonstrates that thrombolysis with tPA alone relies on limited interface contact, leading to obvious limitations in its efficacy and making it difficult to achieve efficient thrombolysis. However, in the experimental group, drug-thrombolytic target contact area gradually increased with channel expansion, speeding up thrombolysis and driving it deeper.

The blood clot dissolution results at 60 min showed that the internal channel of the thrombus in the experimental group continued to dissolve and further expand. Notably, slight collapse occurred at both ends of the thrombi in the experimental group, indicating that some drug diffused to the opposite side via the internal channel and reacted with the thrombus interface. Therefore, a continuous increase in the thrombolytic rate was observed after 60 min. Subsequently, the thrombolysis characterization results from 90 to 120 min demonstrated the continuous dissolution process of the blood clot in the experimental group following the expansion of the penetrating channel. Therefore, During this phase, further disintegration occurred at both ends of the thrombus in the experimental group, with a significant difference in thrombus length relative to the control group. Penetration of the thrombus by MPCs delayed the saturation of the tPA thrombolytic rate, with the saturated average thrombolytic rate of the MPC group being 1.76-fold that of the control group. This result indicates that the channel formation and an increase in surface area is the dominant contributor to the enhancement of thrombolysis (Diamond and Anand, 1993; Bannish et al., 2017).

#### 3.5.2 Relationship between thrombolytic efficiency of PMF-driven MPCs and penetration number

To investigate whether the multiple reciprocating penetration actions of the MPCs can accelerate the thrombolytic effect of tPA after penetration, thrombolysis experiments with the number of penetrations as a variable were conducted. Figure 5C shows the typical morphological changes of the thrombus in the pipeline after 60 min of drug treatment following different numbers of MPC-mediated penetrations. The results indicate that adjusting the number of penetrations *N* significantly alters the size of the penetration channels during the drug thrombolysis phase. Moreover, the area of the penetrating channels within the thrombus increases with the number of MPC reciprocating penetrations. Quantitative analysis at the 60-min mark (Figure 5D) further reveals that when *N* increases from 5 to 15, the drug thrombolysis rate rises with statistically significant differences, while no significant change is observed when *N* increases from 15 to 20. This phenomenon indicates that when the number of penetration cycles exceeds a specific threshold, the effective working region of the MPCs becomes spatially constrained, leading to a state of diminishing returns. In contrast, the thrombolysis rate achieved by tPA alone is notably lower than that in all pulsed penetration groups, with the maximum increase in the thrombolysis rate in the pulsed penetration groups reaching more than twice that of the control group. Therefore, the multiple reciprocating penetration actions of the MPCs gradually expand the initial internal channels within the thrombus, thereby significantly accelerating the thrombolytic effect of tPA post-penetration.

#### 3.5.3 Evaluation of MP retention after PMF-driven MPC penetration and magnetic retrieval

**FIGURE 6:**
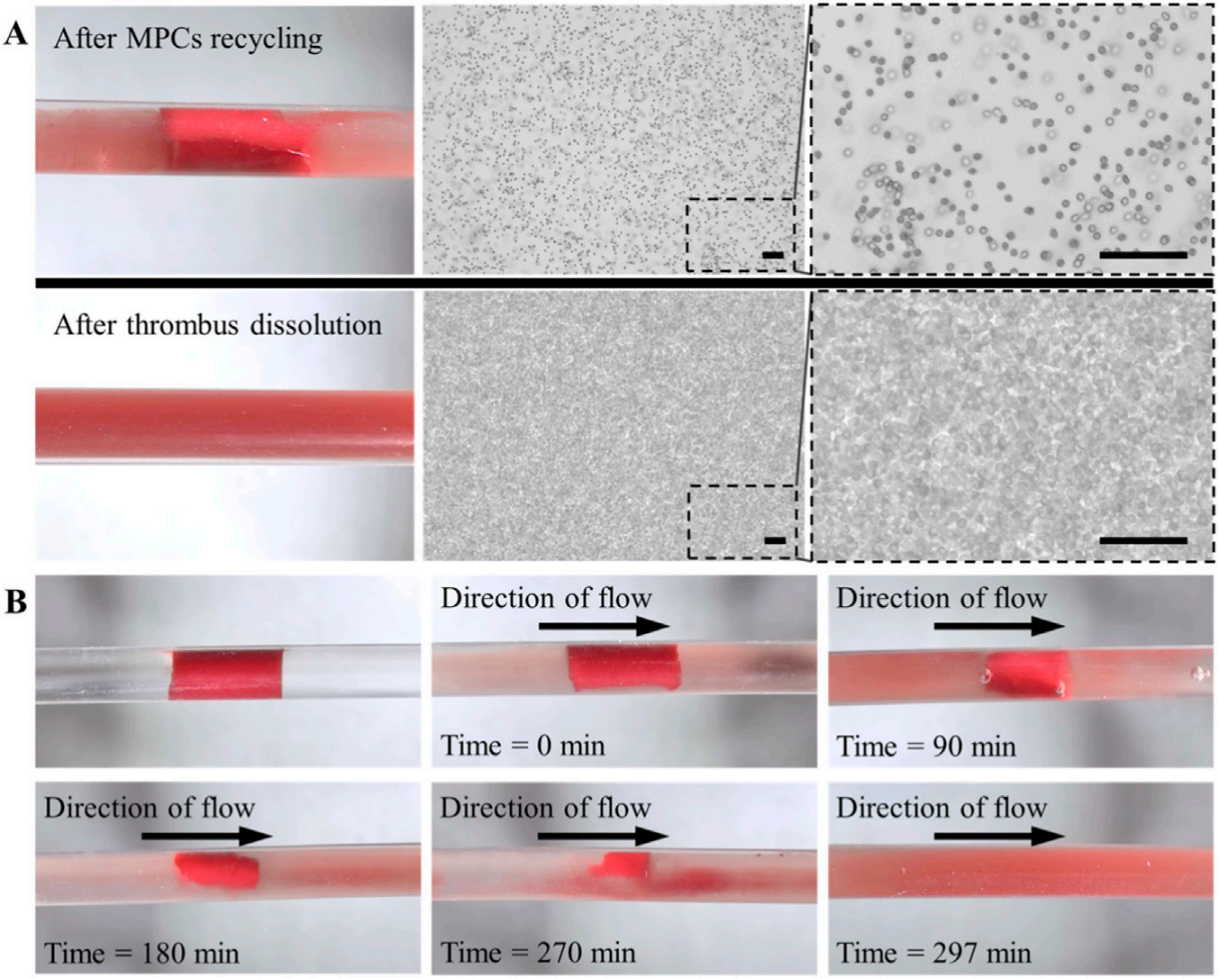
Post-penetration MPs retrieval and dynamic-flow thrombolysis after PMF-driven MPC penetration. **(A)** Post-treatment thrombus morphology and residual MPs observation after MPC penetration and magnetic retrieval. Scale bars = 50 μm. **(B)** CCD images showing thrombus morphological changes under continuous tPA perfusion after PMF-driven MPC penetration and magnetic retrieval. The thrombus continued to dissolve under sustained tPA perfusion and was completely dissolved after 297 min.

As shown in Figure 6A, after MPCs penetration and external magnetic retrieval, no obvious magnetic particle distribution or retention was observed on the thrombus surface. In addition, no detectable magnetic particles were observed in either the liquid released around the thrombus after penetration or the lysate collected after complete thrombus dissolution. These results indicate that PMF-driven MPC penetration followed by external magnetic retrieval did not cause detectable MPs retention within the thrombus.

#### 3.5.4 Dynamic-flow thrombolysis following PMF-driven MPC penetration and magnetic retrieval

Figure 6B shows the morphological changes of the thrombus under dynamic-flow conditions. The results demonstrated that PMF-driven MPCs could still stably penetrate the thrombus and open an internal channel under continuous perfusion. After penetration, the MPs were retrieved using a permanent magnet, and the thrombus continued to dissolve gradually under tPA perfusion. Complete thrombus dissolution was achieved after 297 min. These results suggest that PMF-driven MPC penetration remained effective under dynamic-flow conditions, while post-penetration magnetic retrieval of MPs may reduce particle retention risk and allow thrombus dissolution to continue under sustained tPA perfusion.

## 4 Discussion

In clinical thrombolytic therapy, catheter-directed drug delivery can increase local drug concentration; however, in distal, deep, or small-caliber vessels, further catheter advancement is often difficult or even infeasible. Under such circumstances, particularly when local blood flow is markedly reduced or temporarily stagnant after vascular occlusion, thrombolytic agents rely mainly on slow diffusion and limited permeation, making it difficult to rapidly reach the thrombus core and achieve efficient recanalization. Against this background, the present study proposes a strategy in which magnetically controlled magnetic particle clusters directly penetrate the thrombus structure, thereby accelerating vascular recanalization while enhancing subsequent drug thrombolysis. In this strategy, transient PMFs are used to regulate MPCs for active thrombus penetration.

Our results suggest that PMF-driven MPC penetrate thrombi in a threshold-dependent manner by overcoming the intrinsic mechanical resistance of the fibrin network. When the magnetic driving force is insufficient, MPCs mainly accumulate at the thrombus surface; once the force exceeds the local yield resistance of the fibrin matrix, progressive penetration into the clot interior can be initiated. This interpretation is supported by previous magnetic-microparticle-based modeling, which estimated that a force of approximately 36 nN acting on embedded magnetic microparticles could induce plastic deformation of a fibrin matrix, corresponding to a fibrin clot yield stress of approximately 4 kPa (Averett, 2014). In the present study, the effect penetration force generated by the microparticle carrier was estimated to be 0.2–2.5 mN. Referring to the cross-sectional area of PMF-assembled MPCs observed in Supplementary Figure S3, the effective MPCs–thrombus contact area *S* was approximated as 0.1 mm^2^. After normalization by this contact area, the corresponding local mechanical stress was estimated as $\sigma =\frac{F_{m}}{S}$ ≈ 2–25 kPa. This stress range is comparable to, and partly exceeds, the reported kPa-level yield stress of blood clots, suggesting that PMF-driven MPCs can overcome the local yield resistance of the thrombus and thereby initiate penetration into the clot interior (Kadri et al., 2019).

Notably, this penetration process does not disrupt the bulk of the clot. High-magnification imaging showed no obvious detached thrombus fragments after thrombolysis, suggesting that PMF-driven MPC penetration mainly induced localized fibrin-network disruption rather than catastrophic thrombus fragmentation (Kim et al., 2014). Furthermore, penetration depth increases approximately linearly with pulse number, indicating that each magnetic pulse contributes incremental mechanical work, which accumulates over successive pulses to produce controllable and progressive thrombus penetration. This process enables complete penetration of a 6 mm thrombus within 10 min and establishes a functional drug diffusion channel. Repeated actuation further enhances thrombolysis, although the effect gradually saturates after approximately 15 cycles. This saturation behavior can be attributed to the reduction in the effective penetration contact area between MPCs and the intact thrombus matrix as the number of PMF cycles increased. During the initial cycles, MPCs repeatedly impacted the thrombus interface and rapidly created a preferential penetration channel. Once this channel was formed, subsequent MPC motion tended to occur partly along the preformed pathway, reducing the probability of direct contact with compact, undamaged thrombus regions. As a result, the incremental increase in penetration area became limited by the reduced effective MPCs–thrombus contact area and the geometric confinement of the vessel model. In addition, the particles dose used in this study was 0.2 mg, corresponding to approximately 0.9% of the initial thrombus mass. This dose was selected to provide sufficient magnetic responsiveness for PMF-driven actuation while avoiding excessive particle loading, and it falls within the dosage range commonly used in related *in vitro* studies of magnetically assisted thrombolysis (Li et al., 2018; Wang et al., 2024).

A key advantage of this strategy is the synergistic regulation of penetration efficiency through the interaction between magnetic field strength and particle mass. Experimental results show that different combinations of these parameters can produce similar penetration performance, allowing flexible optimization under practical constraints such as coil heating, power consumption, and biosafety considerations related to particle dosage. The observed isoforce and penetration-efficiency contours therefore provide direct guidance for magnetic thrombolysis system design.

More importantly, PMF-driven MPC penetration fundamentally alters intrathrombus transport. Unlike tPA treatment alone, which is largely limited by surface-dominated fibrinolysis and restricted diffusion into the dense fibrin network, MPC penetration creates internal transport channels, enlarges the effective drug–thrombus interface, and enables tPA to access the clot interior more directly. Consequently, thrombolytic saturation is delayed, and thrombolytic efficacy is enhanced by more than two-fold compared with tPA alone *in vitro* model, consistent with the critical role of intrathrombus transport in fibrinolysis (Diamond and Anand, 1993; Bannish et al., 2017). Other physical-field-assisted strategies also improve thrombolysis by enhancing mass transfer or local clot disruption; for example, ultrasound combined with tPA and microbubbles increased clot-lysis rates by 8%–130% under different microbubble concentrations (Goel et al., 2020), while targeted NIR-triggered nanotherapy achieved *in vitro* thrombolysis rates of up to 85% (Song et al., 2023). In comparison, the present strategy combines rapid mechanical penetration and internal-channel formation providing a potentially complementary approach for integration with catheter-directed or other field-assisted thrombolytic modalities.

In addition to thrombolytic performance, preliminary cytocompatibility was evaluated to assess the biological safety of the magnetic particles. NIH/3T3 cells were selected as a representative fibroblast model commonly used for biomaterial cytotoxicity screening, whereas HUVECs were included to better reflect the potential response of vascular endothelial cells to particles used in thrombolytic applications (Safi et al., 2010; 2011; Wen et al., 2019). CIP@SiO_2_ particles showed improved cytocompatibility compared with unmodified CIP particles, suggesting that SiO_2_ modification reduced the inhibitory effect of the particles on cell viability. Porcine blood was used to prepare the thrombus models because it is readily available in sufficient volume and has been widely employed as a large-animal surrogate for human blood in coagulation and fibrinolysis studies (Olsen et al., 1999; Kessler et al., 2011). This model enabled the reproducible preparation of both red and white thrombi and facilitated controlled evaluation of PMF-driven MPC penetration and thrombolytic enhancement. Nevertheless, validation using thrombi prepared from fresh human donor blood would further strengthen the translational relevance of this strategy.

Beyond cytocompatibility and model relevance, reliable particle recovery after treatment is essential for translational safety. In the present experiments, no detectable residual MPs were observed in the tube, thrombus-derived liquid, or post-lysis samples after MPC penetration and retrieval using an external NdFeB permanent magnet. These findings provide preliminary evidence that post-penetration magnetic recovery can effectively remove MPs from the treatment region and may reduce the risks of particle retention and downstream migration. In future catheter-assisted applications, MPs could be delivered proximally, assembled into MPCs for thrombus penetration, and subsequently removed through catheter aspiration or magnetic recollection. Such a closed-loop strategy would be consistent with emerging reports of catheter-assisted magnetic particle or microrobotic retrieval and could substantially improve the overall safety profile of the technique (Wang et al., 2024; Xu et al., 2025). Further studies using *in vivo* vascular models can support the establishment of a clinically applicable closed-loop framework for PMF-driven MPC-mediated thrombolysis.

## 5 Conclusion

We successfully developed a PMF-regulated MPC system for controllable thrombus penetration and enhanced thrombolysis. The penetration efficiency of MPCs could be effectively regulated by adjusting the magnetic flux density, particle mass, and pulse waveform parameters, enabling controlled interaction with dense thrombus structures. Under PMF actuation, magnetic particles dynamically assembled into clusters that amplified local magnetic forces and induced cumulative, threshold-dependent deformation of and penetration into thrombi. This penetration process created internal transport pathways, improved drug diffusion and subsequent enzymatic fibrinolysis, and resulted in a thrombolysis rate more than twice that achieved with drug treatment alone.

The safety and dynamic-flow experiments further supported the feasibility of this strategy. CIP@SiO_2_ particles remained relatively dispersed in whole blood without PMF actuation and did not spontaneously form large clusters. Hemolysis remained approximately 2%–3% over the tested concentration range of 0–5000 μg/mL. After MPCs penetration, MPs were retrieved using an external NdFeB permanent magnet, and no residual MPs were detected by microscopic observation. Moreover, MPCs retained their penetration capability under continuous perfusion at 10 mL/hr, and thrombolysis continued after MPs retrieval, with complete thrombus dissolution achieved after 297 min. Collectively, as a simplified and controllable magnetic actuation strategy, the proposed MPC system offers a potentially practical alternative to more complex microrobotic or multimodal thrombolytic approaches.

## Data Availability

The original contributions presented in the study are included in the article/Supplementary Material, further inquiries can be directed to the corresponding authors.
