# Supplementary material for: Magnetic particle clusters with tunable thrombus penetration for enhanced thrombolysis
Source: Front Bioeng Biotechnol. 2026 Aug 5;14:1852074. doi: 10.3389/fbioe.2026.1852074 (PMC13485900; doi:10.3389/fbioe.2026.1852074)
Supplement: Supplementary file 1 [file Supplementaryfile1.docx]

Supplementary Material

# Pulsed Magnetic Field Driving Force Model

In this study, the PMF-induced morphological remodeling can be explained by the magnetic dipole interaction between particles and the magnetic force acting on the cluster. Under the linear magnetization assumption, the effective magnetic moment of a particle is given by:

$$m_{\text{eff}}=\frac{V\chi B}{\mu_{0}}$$

where *V* is the particle volume, *χ* is the magnetic susceptibility, *B* is the magnetic flux density, and *μ*_0_ is the vacuum permeability. Therefore, increasing the PMF amplitude directly enhances the effective magnetic moment of the particles. The magnetic dipole interaction force between two magnetized particles can be written as:

$$F\left(r_{ij},\theta\right)=\frac{3\mu_{0}m_{i}^{2}}{4\pi r_{ij}^{4}}\left(1-3\cos^{2}\theta\right)$$

where ***r****_ij_* is the interparticle distance and *θ* is the angle between the interparticle axis and the magnetic field direction. When particles are aligned along the magnetic field direction, *θ*=0°, and the interaction becomes attractive, promoting chain-like assembly along the field. In contrast, when the interparticle axis is perpendicular to the field direction, *θ*=90°, the interaction does not favor axial chaining. Therefore, PMF driving tends to guide the particles to assemble preferentially along the magnetic field direction.

In addition to the interparticle dipole interaction, MPCs are subjected to a magnetic force in a non-uniform magnetic field, which can be expressed as:

$$F_{\text{m}}=(m_{\text{eff}}\cdot\nabla)B$$

By substituting the effective magnetic moment, the magnetic force becomes:

$$F_{\text{m}}=\frac{\chi m_{\text{p}}}{\rho\mu_{0}}(B\cdot\nabla)B$$

This equation indicates that the magnetic driving force is determined by both the magnetic field amplitude and its spatial gradient. As the PMF amplitude increases, both the interparticle dipole attraction and the cluster-scale magnetic driving force are enhanced, facilitating particle assembly, chain merging, and structural compaction.

# Supplementary Figures

**Supplementary Figure 1.** Number-based particle size distribution of CIP@SiO₂ particles obtained from SEM image analysis, with a mean particle diameter of 3.27µm.

**Supplementary Figure 2.** Elemental mapping images of CIP@SiO₂ particles.

**Supplementary Figure 3.** Macroscopic agglomeration morphology of MPCs under PMF.

**Supplementary Figure 4.** Cell viability of NIH/3T3 cells and HUVECs after co-culture with CIP and CIP@SiO₂ particles. (a,b) Cell viability of NIH/3T3 cells after 24h co-culture with CIP and CIP@SiO₂ particles, respectively. (c,d) Cell viability of HUVECs after 24h co-culture with CIP and CIP@SiO₂ particles, respectively. Data are presented as mean ± SD (*n*=3, *P < 0.05, **P < 0.01, and ***P < 0.001).

**Supplementary Figure 5.** Coil temperature variation under different amplitudes of PMF.

**Supplementary Figure 6.** Hemolysis evaluation of CIP@SiO₂ particles under PMF treatment.

**Supplementary Figure 7.** Initial distribution of CIP@SiO₂ particles in whole blood without PMF. Scale bar = 100 μm.

**Supplementary Table 1.** Quantitative EDS analysis of elemental composition in CIP@SiO₂ particles.

| Element | Weight % | Atomic % |
| --- | --- | --- |
| C | 10.73 | 30.30 |
| N | 0.91 | 2.22 |
| O | 9.03 | 19.14 |
| Si | 0.25 | 0.30 |
| Fe | 79.09 | 48.05 |
